# Bioguided Fraction and Isolation of the Antitumor Components from* Phyllanthus niruri* L.

**DOI:** 10.1155/2016/9729275

**Published:** 2016-09-29

**Authors:** Zhi-Zhong Zheng, Liang-Hua Chen, Shao-Song Liu, Yuan Deng, Guo-Hua Zheng, Yue Gu, Yan-Lin Ming

**Affiliations:** ^1^Xiamen Key Laboratory for Plant Introduction, Quarantine and Natural Products, Xiamen Overseas Chinese Subtropical Plant Introduction Garden, Xiamen 361002, China; ^2^Fujian Key Laboratory of Physiology and Biochemistry for Subtropical Plant, Fujian Province Institute of Subtropical Botany, Xiamen, China; ^3^College of Chemical Engineering, Huaqiao University, Xiamen, Fujian 361021, China

## Abstract

*Phyllanthus niruri* L., a well-known medicinal plant, has been used as a folk antitumor remedy in the worldwide scale. However, the antitumor components in* P. niruri* have not been reported. In order to verify the antitumor components of* P. niruri* and the plants which have the high content of these components, we isolated the antitumor components with bioguided fraction and isolation, by different chromatographic methods from the ethyl acetate fraction of* P. niruri.*, and identified them as ethyl brevifolincarboxylate and corilagin by ^1^H-NMR, ^13^C-NMR, 2D-NMR, and mass spectrometric analyses. Cell cytotoxicity assays showed that corilagin has broad-spectrum antitumor activity, a better antitumor potential, and lower toxicity in normal cells. Besides, the coefficient of drug interaction (CDI) of 10 *μ*M corilagin and 20 *μ*M cDDP reached up to 0.77, which means corilagin can promote the antitumor activity of cDDP. Furthermore, by the extensive screening among 10 species of plants reported to contain corilagin, we found that* Dimocarpus longan* Lour. has the maximum content of corilagin. In conclusion, corilagin is the major active antitumor composition in* P. niruri.* L. on HCC cells and has high content in* D. longan*.

## 1. Introduction

*Phyllanthus niruri* L. (*P. niruri*) is a member of the Euphorbiaceae family that consists of over 600 species of shrubs, trees, and annual or biennial herbs [1]. It usually occurs as a winter weed throughout the tropical and subtropical areas including China, India, and America.* P. niruri* is used as medicated food to nurse people's health, especial hair [2], intestine [3], liver [4], and kidney [5] problems and has provoked much interest. In the folk,* P. niruri* has been claimed to present beneficial therapeutic effects in treating kidney and gallbladder stones, liver related diseases such as jaundice and liver cancer, viral infections such as hepatitis and tuberculosis, malaria, diabetes, and fever [6, 7].

In recent years, systematic studies on the constituents and medicinal effects of* P. niruri* have been reported gradually.* P. niruri* has been found to exhibit antitumor [8], antiviral [4], antioxidant [9], anti-inflammatory [10], and antidiabetic [11] activities and radiation protection [12]. Particularly in antitumor activity,* P. niruri* extract shows potential in reducing chemically induced skin papillomas by enhancing antioxidant defense systems [13] and shows potential in the management of a two-stage skin carcinogenesis model in mice. The methanol extracts of* P. niruri* exhibited a 59.5% inhibition of rat aortic vascular growth and showed a significant decrease of 37.9% in the tube formation assay involving human umbilical vein endothelial cells (HUVECs) on Matrigel [14].

Hepatocellular carcinoma (HCC) is one of the deadliest cancers in the world and was ranked the second cause of cancer death [15]. The main treatment of HCC, chemotherapeutic treatment, suffers from increasing resistance responses [16, 17] among patients to existing drugs, lack of wider activities, and selectivity. To overcome these, the development of novel anticancer agents is an immense demand. Previous reports had implied that* P. niruri* is a candidate folk medicine [13, 18, 19]. However, most of previous studies* in vitro/vivo* just focused on the crude extracts from* P. niruri* [3, 20]. The major active compounds in the crude extracts remain unknown. Little work has been performed to determine which active constituents from* P. niruri* have antitumor activity. Fujiki indicated that, to investigate* P. niruri*, a useful and practical method for screening preventive cancer agents is needed [21].

Thus, due to the presence of various medicinal properties, the present study was designed to isolate, screen, and identify the structure of really active components from* P. niruri* and determine their biological activity on HCC.

## 2. Materials and Methods

### 2.1. Plant Material

Whole* P. niruri* plants were collected from Gulangyu Islet, Fujian province, China, in October 2009 and identified by Professor Yong-Tian Zhang, Fujian Province Institute of Subtropical Botany, China. A voucher specimen (YZY20091026) was deposited at Xiamen Overseas Chinese Subtropical Plant Introduction Garden, China.

### 2.2. Chemicals and Reagents

All solvents and chemicals used for extraction in this study were of analytical grade and obtained from Tianjin Reagent Company (Tianjin, China). Materials for column chromatography included polyamide resin (100–200 mesh, Zhejiang Tetracarboxylic Biochemical Plastics Ltd., China) and Sephadex LH-20 (Amersham Pharmacia Biotech, Sweden). The SMMC7721, Bel7402, MHCC97-H, HepG2, OC316, SGC7901, QBC939, and Chang-liver normal liver cell line were obtained from the Cell Bank of the Chinese Academy of Sciences (Shanghai, China). RPMI 1640 medium and Dulbecco's Modified Eagle Media (DMEM) were obtained from Gibco (Grand Island, NY). The 3-(4,5-dimethylthiazol-2-yl)-2,5-diphenyltetrazolium bromide (MTT) reagent was obtained from Sigma (St. Louis, MO). Trypsin, Leibovitz's L-15 medium, fetal bovine serum (FBS), and penicillin/streptomycin solution (200x) were obtained from Mediatech Inc. (Herndon, VA).

### 2.3. Chromatograph Conditions

The chromate column was Waters XBridge™ Shield RP18 (4.6 mm × 250 mm, 5 *μ*m); DAD detector detection wavelength: 280 nm; column temperature: 30°C; mobile phase: Acetonitrile (A) 0.1% KH_2_PO_4_ (B); flow speed: 1 mL/min; injection volume: 20 *μ*L. Elution procedures are as follows: 0–10 min, *V*(A) : *V*(B) = 15 : 85; 10–15 min, *V*(A) :* V*(B) = 20 : 80; 15–20 min,* V*(A) :* V*(B) = 25 : 75.

### 2.4. Standard Curve

We weighed 2.5 mg corilagin precisely, dissolved it in a 10 mL measuring flask with 50% methanol, and prepared a standard solution whose concentration is 0.25 mg/mL; diluted corilagin standard solution into 0.025, 0.050, 0.075, 0.100, and 0.125 mg/mL; injected the diluted solution into HPLC and then determined absorption spectrums. We could get the linear relation from peak area (*Y*) and concentration (*X*).

### 2.5. Preparation of Test Solution

Five g sample powder was extracted with 25 mL of 95% ethanol at room temperature for three times (12 h each time). The solvent was evaporated in vacuo, and the dried ethanol extract was dissolved in 50% methanol (100 mL) and then we filtered it with 0.45 *μ*m membrane; the subsequent filtrate was the test solution.

### 2.6. General Procedure

Compounds were purified on polyamide column, D101 MCI gel and Sephadex LH-20 column, analyzed qualitatively by TLC and quantitatively on ODS-C18 column (150 × 4.6 mm) with a UV detector and MeOH-water (7 : 3) as mobile phase, and determined by HPLC. IR spectra were obtained with a Nicolet AVAIAR 360 FT-IR spectrometer with KBr pellets. The^ 1^H-, ^13^C-, and 2D-NMR spectra were recorded on Bruker AV-400 spectrometers at room temperature (*δ* in ppm, *J* in Hz). Mass spectrometry was carried out on a VG AutoSpec-3000 spectrometer or a Finnigan MAT 90 instrument. EI-MS measurements were carried out on an LCQ-Finnigan instrument at room temperature.

Column chromatography was performed with polyamide resin (100–200 mesh) and Sephadex LH-20. Fractions were monitored using TLC, and spots were visualized by heating silica gel plates or spraying with 5% H_2_SO_4_ in ethanol.

### 2.7. Extraction, Fractionation, and Separation of Antitumor Compounds

The dried and powdered whole* P. niruri* (3 kg) were extracted with 75% EtOH (3 × 5 L). The 75% EtOH extract was combined and evaporated under reduced pressure to yield a residue. Then, it was suspended in water and partitioned with petroleum ether, CHCl_3_, EtOAc, and* n*-BuOH (3 : 1,* v/v*, for 3 times), resulting in five fractions: petroleum ether fraction (Fr.1, 95 g), CHCl_3 _fraction (Fr.2, 58 g), EtOAc fraction (Fr.3, 48 g),* n*-BuOH fraction (Fr.4, 113.7 g), and water fraction (Fr.5, 62 g). After that, the cytotoxicity of five fractions on HCC cell lines was determined by MTT assay. Fraction showing the highest cytotoxicity was further fractionated by column chromatography each time.

**Figure 1:**
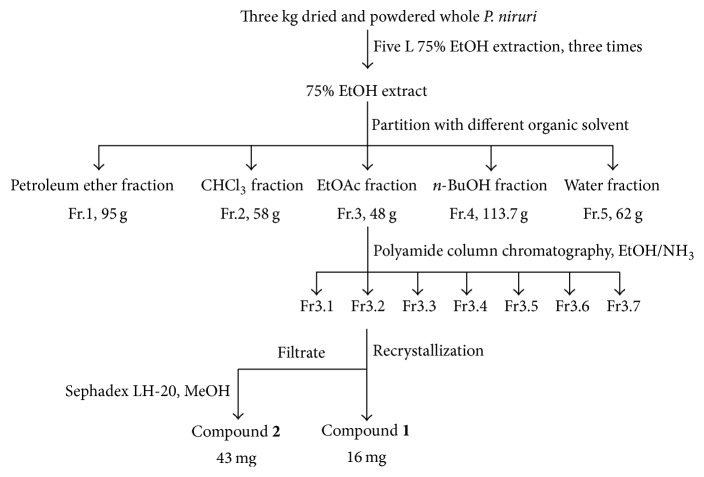
Scheme of bioguided fractionation and isolation of compounds from* P. niruri*.

Fr.3 was separated by polyamide column chromatography using a gradient of H_2_O-EtOH (10 : 0, 8 : 2, 6 : 4, 4 : 6, 2 : 8, and 0 : 10) and then 3.5% NH_3_-H_2_O to yield seven fractions (Fr.3.1–Fr.3.7). For Fr.3.2, the solvent was allowed to slowly evaporate, causing the compound to precipitate, which was then collected by filtration and recrystallized to yield yellow, diamond, flake crystallization (compound** 1**, 12.6 mg). The filtrate was then separated by chromatography on a Sephadex LH-20 column and eluted with MeOH to obtain compound** 2** (43.0 mg) (Figure 1).

### 2.8. Cell Culture

Cells were cultured in RPMI 1640 (SMMC7721, Bel7402, SGC7901, OC316, and QBC939) or DMEM (Chang-liver, HepG2, and MHCC97-H) supplemented with 10% FBS, penicillin (100 U/mL), and streptomycin (100 *μ*g/mL) in a humidified incubator aerated with 5% CO_2_ and 95% air at 37°C.

### 2.9. Cell Cytotoxicity Assays

The effect of constituents on the growth of SMMC7721, Bel7402, and Chang-liver cells was determined using the MTT assay. Briefly, 5000 cells per well in 96-well microtiter plates were treated with corilagin at 0, 10, 20, 40, 80, 120, and 160 *μ*g/mL or DMSO (0.1%,* v/v*) for 48 h. Then, 20 *μ*L of MTT (5 mg/mL) was added to each well, and the plates were incubated for additional 4 h at 37°C. The medium was replaced with DMSO to dissolve the formazan produced from MTT by viable cells. Absorbance at 492 nm is proportional to the live cell count, and cell survival was expressed as the absorbance of MTT-treated cells relative that of DMSO-treated controls.

### 2.10. Drug Combinations

As cell cytotoxicity assays, SMMC7721 was treated with corilagin (5, 10, and 20 *μ*M) and cis-platinum (cDDP, 10, 20, and 40 *μ*M) alone or together with SMMC7721 for 48 h. The coefficient of drug interaction (CDI) was calculated as follows: CDI =* AB*/(*A* ×* B*). According to the absorbance of each group,* AB* is the absorbance of the combination group;* A* or* B* is the absorbance of the single drug group. Thus, CDI value <1, =1, or >1 indicates that the drugs are synergistic, additive, or antagonistic, respectively. CDI value <0.7 indicates that the drugs are significantly synergistic. Thus, CDI was used to analyze effects of drug combinations [22].

### 2.11. Statistical Analysis

All experiments were repeated at least three times, and each experiment was performed in duplicate. The data are expressed as the mean ± SD. Statistical analysis was performed using Student's *t*-test.

## 3. Results

### 3.1. Antitumor Activity of Components Extracted from* P. niruri*

Five fractions, petroleum ether fraction (Fr.1), CHCl_3_ fraction (Fr.2), EtOAc fraction (Fr.3),* n*-BuOH fraction (Fr.4), and water fraction (Fr.5), were extracted from 75% EtOH extract of* P. niruri*. We estimated the antitumor activity of five fractions by MTT assay and found that the IC_50_ values of the Fr.3 and Fr.4 were lower than others (Table 1). So Fr.3 and Fr.4 will be further fractionated by column chromatography.

Fr.3 was further separated by polyamide column chromatography using 1 L H_2_O-EtOH (10 : 0, 8 : 2, 6 : 4, 4 : 6, 2 : 8, and 0 : 10) and 3.5% NH_3_·H_2_O, respectively, to yield seven fractions (Fr.3.1–Fr.3.7). MTT assay showed that the IC_50_ value of Fr.3.2 was the lowest one (Table 1). So Fr.3.2 will be further fractionated.

Fr.3.2 was dissolved in 1 mL EtOH at 50°C, added in 1 mL, and at rest overnight. Compound** 1** was recrystallized and supernatant was eluted by Sephadex LH-20 column with 1 L 50% MeOH. Compound** 2** was separated and purified. The IC_50_ values for Chang-liver, SMMC7721, and Bel7402 cells were 73.49 ± 19.18, 35.89 ± 3.99, and 24.31 ± 0.66 *μ*g/mL for compound** 1** and 93.35 ± 11.99, 14.82 ± 1.00, and 15.52 ± 4.25 *μ*g/mL for compound** 2**, respectively (Table 1). So compound** 2** maybe is the best antitumor compound in the Fr.3.

### 3.2. Spectrometric Identification of Two Compounds

**Figure 2:**
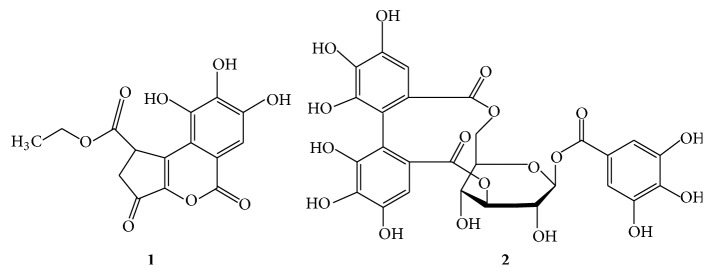
Chemical structures of isolated constituents from* P. niruri*.

Chromatographic purification of* P. niruri* led to the isolation of compounds** 1 **and** 2**. Structural identification was performed by comparing the ^1^H- and ^13^C-NMR spectra with those reported in the literature. The structures of the components identified are presented in Figure 2, and their spectral details are shown below. These compounds were identified as ethyl brevifolincarboxylate** (1) **and corilagin** (2)**.

*Ethyl brevifolincarboxylate* ***(1)***. ^1^H-NMR (600 MHz, DMSO-*d*_6_) *δ*: 1.19 (3H, t,* J* = 7 Hz, -OCH_2_CH_3_); 4.05 (2H, q,* J* = 7 Hz, -OCH_2_CH_3_); 2.44 (1H, dd, *J*_*AB*_ = 18 Hz, *J*_*AX*_ = 2 Hz, H-10); 2.98 (1H, dd, *J*_*AB*_ = 18 Hz, *J*_*BX*_ = 7 Hz, H-10); 4.40 (1H, dd, *J*_*BX*_ = 7 Hz, *J*_*AX*_ = 2 Hz, H-9); 7.29 (1H, s, Ar-H). ^13^C-NMR (126 MHz, DMSO-*d*_6_): *δ*_C_ 145.77 (C=2), 138.49 (C=3), 114.93 (C=3a), 143.63 (C=140.20), 149.60 (C=6), 108.02 (C=7), 112.98 (C=7a), 160.11 (C=8), 40.69 (C=9), 37.01 (C=10), 193.02 (C=11), 171.99 (C=O), 60.56 (C=12), and 13.86 (C=13).

*Corilagin* ***(2)***. ^1^H-NMR (500 MHz, DMSO-*d*_6_): *δ*_H_ 7.01 (2H, s, H-2′ and H-6′), 6.55 (1H, s, H-6′′), 6.49 (1H, s, H-6′′′), 6.20 (1H,* d*,* J* = 7.5 Hz, H1), 4.58 (1H, br s, H-3), 4.35 (1H, t,* J* = 8.5 Hz, H-5), 4.22 (1H, br s, H-4), 4.21 (1H, dd,* J* = 11.0, 8.5 Hz, H-6), 3.94 (1H, dd,* J* = 11.0, 8.5 Hz, H-6), and 3.87 (1H, d,* J* = 7.1 Hz, H-2); ^13^C-NMR (126 MHz, DMSO-*d*_6_): *δ*_C_ 167.2 and 167.5 (C=O), 145.6 (C-3′′′), 145.3 (C-3′′), 144.4 (C-5′′′), 144.8 (C-5′′), 135.9 (C-4′′′), 136.0 (C-4′′), 116.0 (C-1′′′), 116.3 (C-1′′), 123.5 (C-2′′′), 124.4 (C-2′′), 106.6 (C-6′′′), and 107.4 (C-6′′); galloyl *δ*_C_ 165.3 (C=O), 146.0 (C-3′ and C-5′), 139.5 (C-4′), 119.2 (C-1′), and 109.5 (C-2′ and C-6′); glucose *δ*_C_ 92.7 (C-1), 78.0 (C-3), 76.8 (C-5), 72.1 (C-2), 64.4 (C-6), and 62.6 (C-4).

### 3.3. Two Compounds Inhibited the Proliferation of Cell Lines

**Figure 3:**
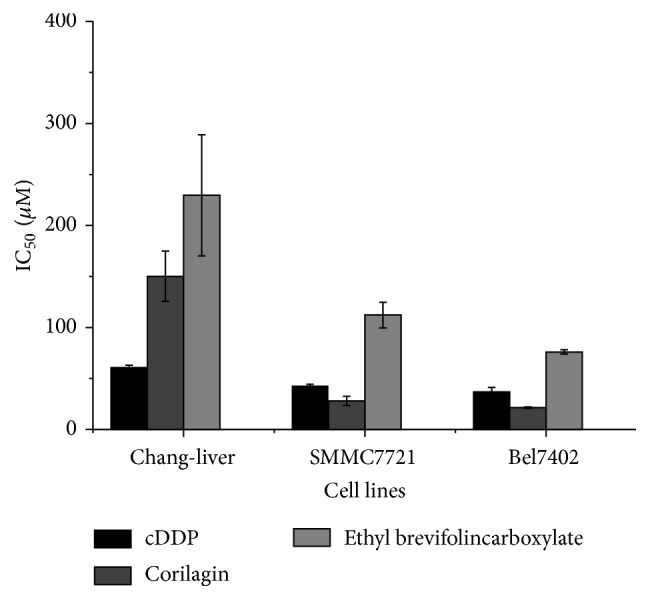
IC_50 _value of two compounds and positive control cDDP on HCC cell lines.

**Figure 4:**
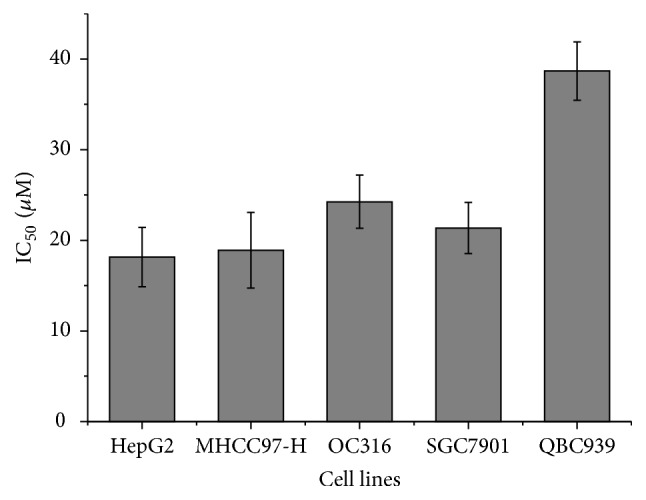
IC_50 _value of corilagin on different cancer cell lines.

To evaluate the cytotoxicity of corilagin, Chang-liver, SMMC7721, Bel7402, HepG2, MHCC97-H, OC316, SGC7901, and QBC-939 cells were treated with increasing concentrations of corilagin (0, 10, 20, 40, 80, 160, and 320 *μ*M) for 48 h, and the cell viability was detected by MTT assay.  Figure 3 shows that corilagin induced a dose-dependent decrease in HCC cell viability and had a lower cytotoxicity to Chang-liver normal cell line (IC_50_  147.16 ± 18.9 *μ*M).  Figure 4 illustrates that corilagin has a strong broad-spectrum antitumor effect.

### 3.4. Corilagin Enhancing Drug Interaction with cDDP on SMMC7721* In Vitro*

**Figure 5:**
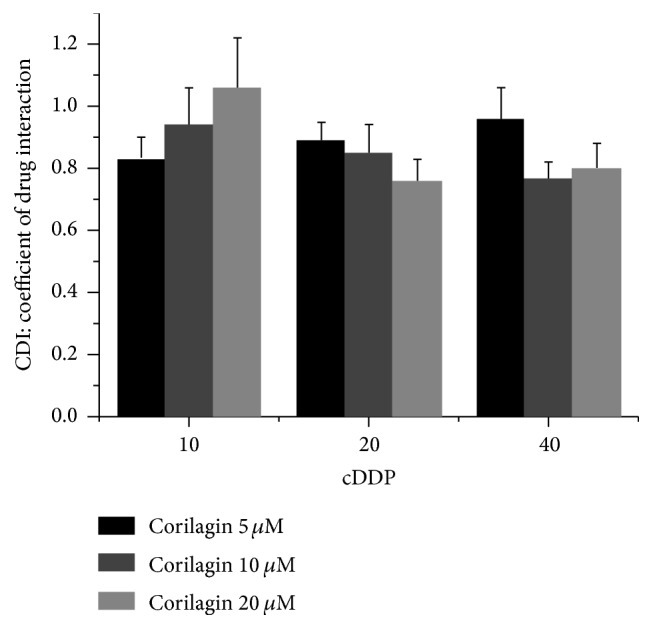
The synergistic antitumor activity of corilagin combined with cDDP on SMMC7721 cells. CDI for the combination treatment of corilagin with cDDP on SMMC7721 cells. Data represent Means ± SE from 3 independent experiments.

Subsequently, we analyzed the nature of the interaction between corilagin and the positive control cDDP using CDI, which quantitatively measures the interaction of two drugs. As shown in Figure 5, corilagin and cDDP yielded synergistic interactions on SMMC7721 across a wide concentration range. The synergistic effect was most prominent when 10 *μ*M corilagin was combined with 20 *μ*M cDDP (CDI = 0.77). This result suggests that corilagin can promote the antitumor activity of cDDP.

### 3.5. The Content of Corilagin in Different Plants

According to the linear relation from peak area (*Y*) and concentration (*X*), we could get the equation of the standard curve: *Y* = 1.03333 + 24742.3*X* (*R* ^2^ = 0.9998). The linear range was 0–125 *μ*g/mL. So we get the data of different corilagin content of plants (Table 2). Results indicated that* Dimocarpus longan* Lour. seed contents the maximum corilagin, up to 643 *μ*g/g (Table 3).

## 4. Discussion

Over the last few years, the* P. niruri* has gained a reputation in folk and traditional medicine, for its multiple healing properties. Previous study tried to induce liver cancer in mice that had been pretreated with a water extract of* P. niruri.* The results indicated the* P. niruri* extract dose dependently lowered tumor incidence, levels of carcinogen-metabolizing enzymes, levels of liver cancer markers, and liver injury markers [23]. So the plant had a prospective antiproliferative and antimetastasis effect against cancer compared to a direct antitumor effect or selective ability to kill a cancer cell.

In this study, with bioguided fraction and isolation, EtOAc fraction significantly inhibits the growth of HCC cell lines. Two compounds were obtained and identified as ethyl brevifolincarboxylate and corilagin. Between them, corilagin had a lower IC_50 _value on HCC cell lines, which indicates that corilagin is the antitumor compound from* P. niruri* [24]. So bioguided fraction and isolation will make sure that the antitumor components from* P. niruri.* can be easier to be separated.

Corilagin, a tannin, was mostly focused on the antioxidant activity [25, 26], whereas the antitumor activity was not much accounted for. In this study, our research suggested that corilagin has better antitumor activity, broad-spectrum antitumor activity, and less toxicity to normal cells and can promote the antitumor activity of cDDP. These data indicate that corilagin can significantly inhibit the growth of HCC cells* in vitro*, which is consistent with the most recent report that corilagin could retard the* in vivo* growth of xenografted Hep3B HCC cells [27] and induce G2/M phase arrest [24]. But the mechanisms of these synergistic interactions need to be further studied.

Besides the favorable antitumor activity, corilagin also has the maximum content in* Dimocarpus longan* Lour. among 10 species of plants reported to contain corilagin, up to 643 *μ*g/g., which means that corilagin has rich sources and is easy to be acquired.

## 5. Conclusions

With bioguided fraction and isolation, we found that* P. niruri.* L. has great inhibiting effect on HCC cells. Corilagin is the major antitumor component that can significantly inhibit the growth of HCC cells and that shows low toxicity to normal cells. So corilagin is the major active antitumor composition in* P. niruri. *L. on HCC cells with growth inhibition.

## Figures and Tables

**Table 1:** The IC_50_ of Chang-liver and HCC cells treated with organic solvent-soluble fractions of *P. niruri. *Data represent Means ± SE from 3 independent experiments.

Components	IC_50_ (*μ*g/mL)		
	Chang-liver	SMMC7721	Bel7402
75% EtOH extract	>100	>100	71.86 ± 0.43

Petroleum ether fraction (Fr.1)	74.63 ± 7.68	68.49 ± 1.96	>100

CHCl_3_ fraction (Fr.2)	>100	84.42 ± 4.97	>100

**EtOAc fraction (Fr.3)**	**69.03 ± 5.33**	**64.92 ± 6.04**	**58.16 ± 3.27**
Fr.3.1	>100	40.72 ± 6.32	>100
**Fr.3.2**	**83.68 ± 3.41**	**21.86 ± 0.60**	**27.80 ± 4.09**
Fr.3.2.1	>100	59.63 ± 18.34	44.20 ± 13.55
Fr.3.2.2	>100	63.39 ± 5.59	67.27 ± 7.23
Compound** 1**	**73.49 ± 19.18**	**35.89 ± 3.99**	**24.31 ± 0.66**
Compound** 2**	**93.35 ± 11.99**	**14.82 ± 1.00**	**15.52 ± 4.25**
Fr.3.3	>100	30.28 ± 0.33	34.88 ± 2.00
Fr.3.4	>100	41.52 ± 0.46	57.41 ± 0.34
Fr.3.5	76.98 ± 6.32	50.73 ± 9.86	56.76 ± 8.70
Fr.3.6	70.70 ± 12.67	>100	>100
Fr.3.7	73.20 ± 6.51	>100	>100

n-BuOH fraction (Fr.4)	96.07 ± 10.94	68.55 ± 6.07	54.25 ± 3.73

Water fraction (Fr.5)	>100	>100	>100

**Table 2:** Experimental samples.

Family	Plant	Part	Acquisition place
Euphorbiaceae	*Phyllanthus urinaria *L.	Whole plant	Gulangyu Islet, China
	*P. tenellus *Roxb.	Whole plant	Gulangyu Islet, China
	*P. debilis* Klein ex Willd.	Whole plant	Gulangyu Islet, China
	*Acalypha australis* L.	Whole plant	Gulangyu Islet, China

Polygonaceae	*Polygonum chinense* L.	Leafy shoot	Gulangyu Islet, China

Saururaceae	*Saururus chinensis* (Lour.) Bail.	Leaf	Gulangyu Islet, China

Combretaceae	*Terminalia catappa *L.	Leaf	Danzhou, China

Sapindaceae	*Dimocarpus longan* Lour.	Fruit rind and seed	Xiamen, China
	*Litchi chinensis* Sonn.	Fruit rind and seed	Xiamen, China

Punicaceae	*Punica granatum *L.	Seed	Xiamen, China

**Table 3:** Content of corilagin from different samples (ND: not detected).

Plant	Content (*μ*g/g)
*Acalypha australis* L.	2.16
*Polygonum chinense* L.	3.76
*Saururus chinensis* (Lour.) Bail.	5.73
*Terminalia catappa *L.	271
*Dimocarpus longan* Lour. (fruit rind)	569
*Dimocarpus longan* Lour. (seed)	643
*Phyllanthus urinaria* L.	619
*P. tenellus *Roxb.	159
*P. debilis* Klein ex Willd.	67.9
*Punica granatum *L. (Seed)	ND
*Litchi chinensis *Sonn. (Fruit rind)	ND
*Litchi chinensis *Sonn. (Seed)	ND
